# Reaching the Unreachable: Intensive Mobile Treatment, an Innovative Model of Community Mental Health Engagement and Treatment

**DOI:** 10.1007/s10597-024-01243-0

**Published:** 2024-03-14

**Authors:** Jana Colton, Roshni Misra, Elise Woznick, Rachel Wiedermann, Anna Huh

**Affiliations:** Center for Urban Community Services, 1789 Madison Avenue, New York, NY 10035 USA

**Keywords:** Community mental health, Harm reduction, Risk management, Engagement, Recovery-oriented, Treatment teams

## Abstract

In this paper we introduce the Intensive Mobile Treatment (IMT) model, which arose from a 2016 New York City initiative to engage individuals who were “falling through the cracks” of the mental health, housing, and criminal justice systems. People who are referred to IMT often have extensive histories of trauma. They experience structural racism and discrimination within systems and thus can present as distrustful of treatment teams. We detail the structure of the program as we practice it at our non-profit agency and outline the psychodynamic concepts that inform our work with challenging populations. We acknowledge IMT’s role in engaging in advocacy and addressing social justice in our work. We also discuss how through this model we are able to both mitigate and tolerate risk in participants with difficult-to-manage behaviors. This is typically a long-term, non-linear process. We address how this impacts the team dynamic as a whole and explain how with long-term, trusting therapeutic relationships, participants can change and grow over time. We also explain the ways in which our non-billing model plays an integral role in the treatment we are able to provide and identify several challenges and areas for program growth. In outlining our model and its methodology, we hope to empower other practitioners to adapt IMT to other settings beyond the New York City area.

## Introduction

Community mental health treatment has made great strides over the last 50 years with the advent of various high intensity community services, including Assertive Community Treatment (ACT), Street Psychiatry or Homeless Outreach, and Modified Therapeutic Communities (Carino & McQuistion, 2022). ACT in particular has been widely referenced as the prevailing mobile treatment modality for community mental health. Providing treatment in the community for patients who had previously been maintained in institutional settings allows for quality of life improvement for many people (Stein & Test, 1980; Bond et al., 2002; Zigura & Stuart, 2000).

Despite these strides, there are a subset of patients whose needs remain unmet by existing treatment models. Gaps in care can arise when patients do not fit into traditional severe mental illness (SMI) categories, when patients are difficult to engage, when patients are highly transient, or when patients experience disruption in care, such as incarceration or long-term hospitalization (Hogan et al., 2008; New York State Office of Mental Health, 2023; Salvara et al., 2013). Among the most frequently incarcerated individuals, there are high rates of SMI, substance abuse, and homelessness, but existing systems are often unable to break the re-incarceration cycle because these individuals never remain in one place long enough to establish care. (Hopkin et al., 2018; MacDonald et al., 2015). Even when mental health treatment is available, the complex psychiatric, substance, medical, and social problems of these individuals can stymie efforts to provide treatment-as-usual. Fragmentation of services, such as housing, hospitals, and substance treatment pose significant barriers to integration and coordination of care, even among some of the highest intensity treatment models (Hogan et al., 2008). As a result, individuals can get stuck between systems in which no one entity assumes clear accountability for their treatment (Smith & Sederer, 2015).

In this paper, we present Intensive Mobile Treatment (IMT), a new model addressing precisely this population that has indeed “fallen through the cracks.” IMT is a robust, much-needed innovation over previous models and, even in early iterations, diverges significantly from existing ACT implementations (Carino & McQuistion, 2022). We will describe the program itself and highlight the unique, collaborative way that it has been implemented and practiced specifically at our organization, which is not necessarily representative of how IMT is practiced elsewhere. Our model is founded on the structure provided by NYC Department of Health and Mental Hygiene (DOHMH); it has then been refined over several years of collaboration between psychiatry, social services, and peer input as well as clinical experience and participant feedback.

The goal of this article is not only to foster understanding of this work, but also to enable other practitioners to utilize aspects of our model for hard-to-reach clients. This model, although founded to address limitations of treatment available in New York City, could potentially be utilized or replicated in other settings with similar gaps in mental health services.

### Intensive Mobile Treatment Criteria and Mission

IMT teams were established in 2016 as an innovative model to provide ongoing treatment to NYC’s hardest-to-reach individuals. It began as a component of the NYC Safe project, which began on the heels of several high-profile violent incidents in NYC (NYC Office of the Mayor, 2015). These incidents were perpetrated by individuals with documented forensic and mental health histories who were not engaged in care. IMT Teams were tasked with providing flexible, multi-disciplinary, long-term, easily accessible treatment to individuals with “a high degree of transience and complex cross-systems involvement” (NYC Health, 2017).

The IMT program has specific criteria. Participants are 18 and older, residents of NYC, and are experiencing homelessness or housing precarity. They have frequent contact with the mental health or substance use systems, as well as the criminal justice system, and they have recent behavior that is unsafe (NYC Health, 2017). Referrals are assigned to IMT teams  through the NYC Single Point of Access (SPOA). Demographic data for participants at our agency are shown in Table 1. Participation is voluntary, but teams are allotted significant time for persistent engagement efforts without the expectation of having to discharge individuals who are reluctant to accept services. Teams are comprised of multidisciplinary staff members, including psychiatric providers, nurses, social workers, case managers, and peer specialists, and are operated by non-profit organizations contracted by the NYC DOHMH.

The IMT funding structure is a crucial component that enables the teams to provide high-quality, person-centered care to a specialized population that meets criteria for the program based on a dynamic assessment of risk as opposed to specific billable diagnostic criteria. IMT teams are fully financed by contracts through the NYC DOHMH and not through Medicaid or other insurance reimbursement. Teams can provide services in a variety of settings that would traditionally be considered "double billing," including hospital stays, rehabs, day treatment programs, and other types of community treatment. By eliminating the constraints of billing, IMT teams can make more nuanced clinical assessments and create customized treatment plans that address the needs of each participant.

This clinical freedom is essential to the ethos of the model, and it empowers the teams to approach cases with flexibility and creativity that is needed for connecting with participants that have not had positive outcomes with other treatment modalities. For example, participant A may require short daily check-ins at their street location, while participant B may benefit more from less frequent but longer therapeutic sessions, and participant C may only be able to tolerate meetings where concrete tasks are being completed. IMT is also flexible and creative in where to meet participants. Team members can engage participants on the street, in the community, at the office, at a hospital, or even in jail. Outreach is assertive, and teams employ a variety of creative strategies to engage the individual in care. A Medicaid-dependent model, such as an ACT team, would be confined by meeting requirements for billable contacts, thus precluding such variations in treatment.

IMT contracts are awarded at $1.1-$1.3 million dollars annually, or roughly $44,400 per participant. While jurisdictions weighing the funding of non-billing IMT teams may consider this a high price for high quality community care, they may also factor that the average cost of incarcerating an individual on Rikers Island falls somewhere around $42,420 per month and hospitalization costs a staggering $108,270 per month (Lander, 2023). The preliminary data on IMT from NYC DOHMH shows that rates of incarceration decline about 30% in the first year participants are engaged with IMT when compared to the 12 months prior to their enrollment (Harrison et al., 2019).

Services are also not time limited. Unlike ACT and other similar services, which are constrained by billing insurance, IMT will follow participants through what would otherwise be interruptions in care, such as incarceration and state hospitalization. This continuity of care reduces participant despair and isolation in such difficult times and allows for the formation of deep and long-lasting relationships. The model keeps treatment teams accountable to each individual participant, in that they are committed to continuing to work with the participant and will not discontinue treatment for what traditionally might be considered dischargeable offenses. The IMT team also serves as a repository of the participant’s social and treatment history as the individual moves through various systems, institutions, and physical locations.

Expectations around the pace of recovery reflect the entrenched challenges of each participant. If the challenges formed over the span of decades, learning something new will likely be a long road. IMT adjusts to and offers what may traditionally be considered extended treatment courses; thus, the pace also allows the team to wait for optimal timing in making interventions, whether they be related to case management, psychopharmacology, or psychotherapeutic interpretations.

The severity of illness of the average IMT participant also means that symptoms and social disengagement may appear intractable for extended periods of time, which traditional treatment teams may be unable to tolerate. Observable behavioral, clinical, or social changes occur quickly for some participants with the consistent safety of IMT, while for others, it is not uncommon to see change only after several years of persistent engagement.

Unlike ACT teams and many other mental health treatment modalities, there are no diagnostic eligibility criteria for IMT, which frees the team from being beholden to a more traditional illness-based model of mental health care. What unites participants is that these diagnoses are often complicated by other comorbid realities, including homelessness, estrangement from family, social isolation, unemployment, medical illness, undocumented legal status, risky behaviors, and involvement in the criminal justice system. It is also common for IMT participants to have experienced ongoing structural racism, cultural bias, and discrimination in these systems.  Table 2 compares the primary structural differences between ACT and IMT

**Table 1:**
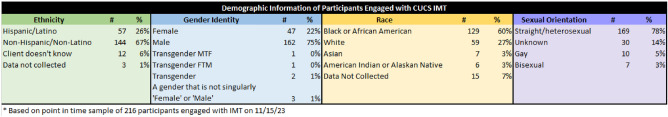
Demographic Data for IMT Participants at CUCS

**Table 2:** Comparison of ACT and IMT

ACT	IMT
Requires specific SMI diagnosis for enrollment	Enrollment criteria is based on assessment of risk and interaction with behavioral, correctional, and shelter systems
Medicaid billing requirement provides guidelines around frequency, length, and type of visits the team provides	Teams are funded through city contracts and use clinical assessment and person-centered recovery plans to determine nature and frequency of visits
Clients cannot be enrolled in other Medicaid-billing programs due to “double billing.”	Participants can attend any other types of programs if desired as teams do not bill Medicaid or other insurance
Clients are transferred to a different team if they leave that team’s “catchment area.”	Emphasis is on long-term therapeutic relationship with the team which follows participants throughout moves and transitions in all 5 NYC boroughs
Discharge may occur due to incarceration or long-term hospitalization	Team may retain a participant on a caseload through a hospitalization or incarceration that lasts 1 year or more

Within this context, the mission of IMT is to help improve the totality of an individual’s life, help find meaning and purpose, and inspire hope. This is achieved via enhanced versions of traditional medical and psychiatric practices and social support, but additionally through creative, flexible, individualized counseling, advocacy (both on an individual and systems level), and support in meeting self-identified needs and goals. Most importantly, the team provides opportunities to form meaningful and secure attachment relationships that promote self-esteem, self-efficacy, and confidence in navigating life's challenges. These relationships, provide a safe and stable foundation from which new patterns of relating to oneself and one’s community can emerge, and through which healing can occur.

### Implementation of Intensive Mobile Treatment

Our organization, Center for Urban Community Services (CUCS), is one of the nation's largest providers of housing and social services to homeless and formerly homeless individuals. Within CUCS is Janian Medical Care, a psychiatric and primary medical care affiliate. Psychiatric care at Janian is conceived of as not simply a consultation service, but rather, an integrated partner to social work across the agency. Thus, in creating our unique model of IMT, we took a similarly integrated approach, which we will now outline.

### Managing Workflow

**Fig. 1:**
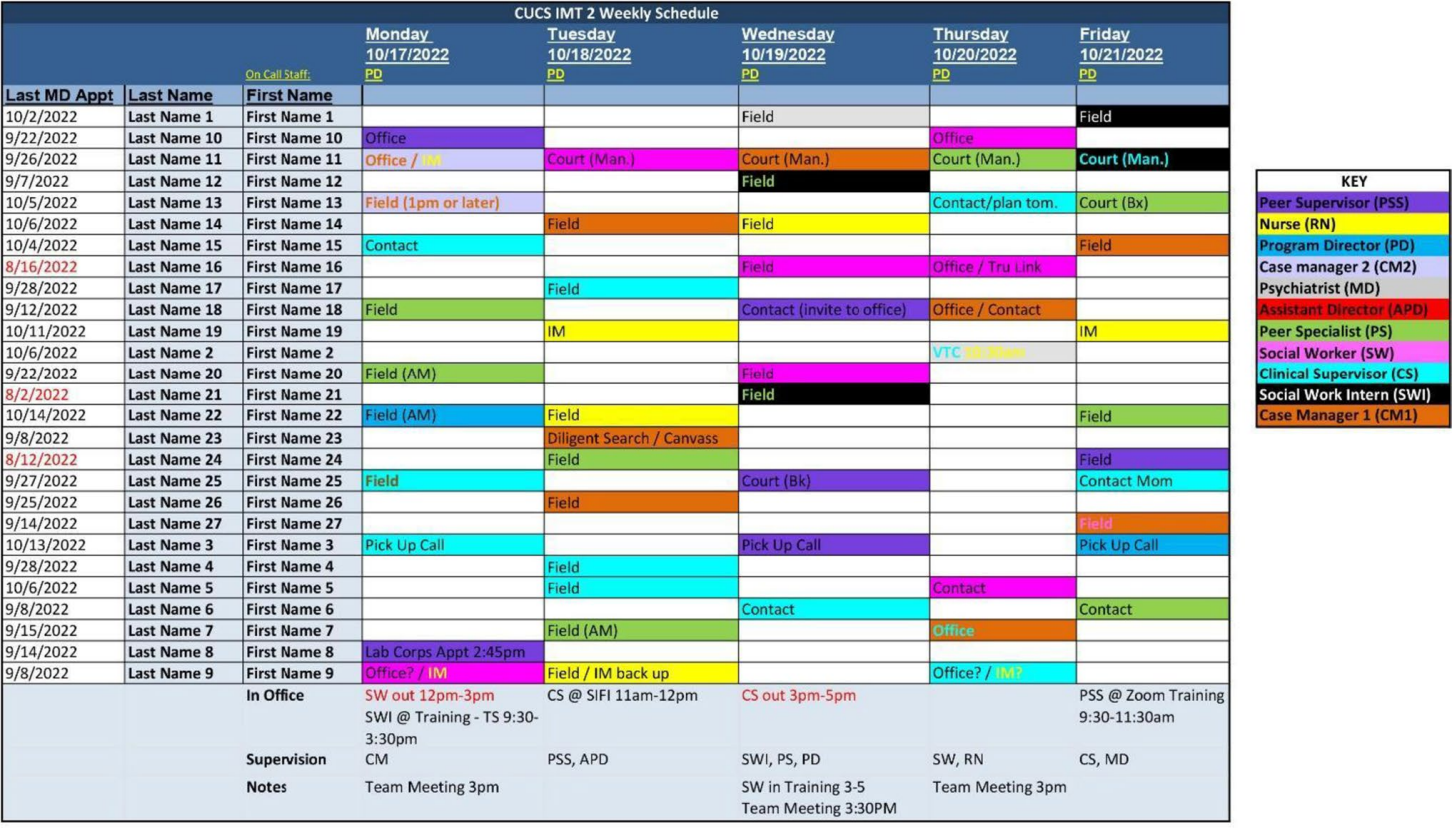
Sample Weekly Schedule

Because the nature of the team’s work is based on rapidly changing participant needs, the IMT program relies on deliberate organization of its workflow to provide structure to its members and participants. Each team has a clinical supervisor who is responsible for overseeing the daily operations of the team. The team maintains a shared calendar where future appointments (e.g., medical and entitlement appointments, court appearances, housing interviews) are recorded. This is updated daily with new information and weekly with an assigned schedule of clinical visits (Fig. 1).

Each IMT team staff gathers for scheduled team meetings 3–5 times per week. These meetings are used to review encounters with participants, discuss ongoing clinical treatment, and process countertransference or other difficult emotions that arise. This is reflected in a “Daily Summary” which is emailed to the team at the end of each day and used as a framework for the team’s communication the following day. During work days, team members email on this thread between visits to keep teammates informed of their whereabouts, share what happened in each visit, and raise new concerns about participants. This method of communication functions as a mechanism for staff to elicit feedback and participate in real-time shared decision making when they are in the field. It also minimizes isolation, generates live clinical conversation, and acts as a safety mechanism for employees.

Field visits are based on participant needs and preferences detailed in the Person-Centered Recovery Plan developed by the team and the participant. They allow for flexibility and may vary greatly in terms of length, location, and frequency. For example, while one participant may receive twice weekly wellness checks in their apartment, another may be outreached at their street location and then accompanied to a medical appointment. Participants are also visited in hospitals, jails, shelters, and street locations. Team members primarily use public transportation to visit participants across all 5 boroughs of New York City, and on occasion, outside of the city as well.

In addition to field visits, each IMT team has regularly scheduled office days with walk-in hours for participants. The office is used as a safe location for participants to work with team members on their goals, apply for benefits, receive psychotherapy, or get rest and warmth if they are living on the street.

### Use of Supervision

All staff that work on IMT teams receive one hour of weekly individual supervision (except for psychiatry, for which supervision occurs twice a month). This time can be used to discuss and review concrete tasks, but is also used for staff to process the reactions and emotions that often surface while engaging and building relationships with IMT participants. These discussions include topics of transference and countertransference, safety planning, and boundary setting. Medical staff also participate in regular supervision with other medically trained colleagues in the agency. Additionally, staff review how team dynamics (e.g., communication, trust, decision making) impact participant treatment.

### Use of Service Dollars

Service dollars are allocated for participant needs and are used when IMT staff incur expenses related to clinically indicated participant treatment goals. Some common applications of service dollars relate to transportation costs, fees for applying for identification documents, medical supplies, and prescription costs for participants who have inadequate insurance. Service dollars are also used as a means to reduce barriers to treatment. For example, the team may purchase a phone for a participant and pay the monthly bill so that they are able to keep in contact. The team is able to budget service dollars to purchase meals for participants during visits, which can foster a casual tone that may be more comfortable for participants who typically balk at traditional “office visit” interview settings. IMT participants often have chronically unmet basic needs, and the team is able to attend to these by using funds to provide items such as clothing or hygiene products. With basic needs met, participants are better able to focus and work on goal-related tasks.

Service dollars are also used for purposes not traditionally implemented by service providers. They are used to build rapport and in other ways that seek to encourage participants’ self-identified goals. For example, a participant may have a goal of improving their family relationships, and the team may assist them with providing funds so they can purchase holiday gifts for their family members. The team also regularly uses funds to normalize and celebrate life milestones such as birthdays, move-ins, and other accomplishments, which further fosters strong rapport with participants. The flexibility surrounding the use of service dollars within IMT is integral to the program, and it has enabled staff to extend the scope of their work with participants in a way that is very effective.

### Shared Caseload

IMT utilizes a shared caseload model. The team supervisor ensures that every staff member is involved in the care of every participant. This arrangement is beneficial to participants in that it allows them to engage with staff who have varying specialties and styles. It enables the team to approach each case from a holistic perspective, as each staff member brings their own perspective and assessment to their formulation. This also provides the team with opportunities to attempt different engagement styles to determine which work best with each participant. The shared caseload also minimizes staff burnout as staff are able to take time away from participants after challenging interactions. In building relationships with every member of the team, participants are able to develop a group transference to the IMT team as a whole, which can be helpful in minimizing disruptions in care in times of staff turnover.

### On call rotation/24-h line

The program operates a 24-h phone line that is available to both participants and outside providers. Salaried staff share the responsibility for covering the line in shifts outlined in an on-call schedule. Program participants are advised that they can access the line and speak with the team member on call if urgent issues arise. The on-call phone number is accessible to city hospital staff through a state-wide database, which enables the team to provide collateral information (medication regimens, diagnostic and historical information) in emergency situations.

### The Role of the Psychiatrist on IMT

The psychiatric provider is an integrated member of the team who provides clinical leadership, advocacy and coordination, in addition to psychiatric treatment and medical co-integration.

Prescribing for IMT participants must be practical and creative, and staff must prescribe medications while acknowledging the participant’s autonomy. This begins with a careful assessment of the risk versus benefit of requiring strict adherence to traditional prescribing, which participants may not tolerate. We begin with evidence-based practices and treatment algorithms but generally must quickly move to find whatever medication within the spectrum of options will be acceptable to an individual. For example, if a primary disorder cannot be treated, the psychiatrist will not hesitate to explore treatment for secondary disorders. If the first line regimen is not accepted, then second line, third line, or even alternative remedies are considered and offered. If a participant cannot or will not take medications on their own, IMT psychiatrists and teams work with residences or other agencies to hand-dispense medications as frequently as possible, within service and safety limits. If a participant is able to take medications independently but refuses to take them as prescribed, we assess the risk of their desired adherence schedule and, within limits, tolerate any associated risks, offer alternatives to compromise, and collaborate to find a frequency or way of taking the meds that is acceptable to the participant. If oral meds are refused, then alternative methods of delivery are explored and offered. Preferences frequently change over time, and thus IMT psychiatrists must respond to those changes as nimbly as the demands may be mercurial. When psychotherapy is indicated, a similarly flexible and creative approach is taken, with the rationale that low fidelity psychotherapy is better than no psychotherapy at all. Through all of this, a non-judgmental attitude is required, as our participants are perceptive of unexpressed judgment and may react by rejecting treatment.

It is worth noting that the IMT psychiatrist role is also not limited to medication management. All participants typically meet with the team psychiatrist immediately after referral to IMT, whether or not they have a stated diagnosis or need for medication and continue to have a minimum of monthly visits throughout their tenure on IMT. Psychiatrists are able to visit with participants through the same shared caseload model as other team members, and they represent a unique specialty and viewpoint on the team and play a valuable part in discussions about participant care and approaches, whether or not medication or traditional psychiatric treatment is involved.

IMT psychiatrists partner with the program directors of the team to provide clinical leadership around developing and implementing treatment plans, promoting evidence based practices on the team, managing clinical crises, identifying areas for staff continuing education and providing clinical trainings.

Clinical coordination is another key component of the psychiatry role, as IMT participants are often involved in various other un-integrated systems, and the psychiatrist works to provide outside providers with clinical histories and to collaborate on treatment plans and care transitions. The IMT psychiatrist also leverages their role and network to help participants access appropriate treatment and supports within various systems and the community.

In partnership with the IMT nurse, IMT psychiatrists have a unique opportunity to provide medical and psychiatric co-integration. Because IMT participants have frequently had poor experiences with the medical establishment, IMT psychiatrists often take on the role of general practitioner and medical care coordinator in addition to providing more traditional psychiatric care. The hard-earned alliance developed between an IMT provider and participant allows participants to engage in medical care that they may not otherwise be able to tolerate in traditional settings. Psychiatrists become trusted partners in care, explaining and guiding participants through medical illness, healthcare, and health systems that are at times confusing and re-traumatizing. Medical needs are often treated empirically or with guidance from internal medicine providers, or in conjunction with a variety of outpatient and urgent care clinics and street medicine teams. Since adherence to medical appointments can be challenging for this population, IMT psychiatrists continue or start medications, and psychiatrists and nurses may be tasked with administering first aid and wound care, treating lice and other infestations, carrying and delivering medical supplies, conducting ad hoc physical exams, and helping to reduce the barriers to attending medical appointments (Table 3).

**Table 3:** The Role of the IMT Psychiatrist

Psychiatric Care	● Encompasses assessments, psychotherapy, and psychopharmacology ● Care must be flexible, creative and practical ● Harm reduction (including MAT, fentanyl test strips) ● Work-arounds for imperfect adherence. Psychotherapy modifications: low fidelity psychotherapy is better than no psychotherapy at all
Clinical Leadership	● Partnership with the PD and APD of the team ● Promote evidence-based practices on the team ● Manage clinical crises ● Identify areas for staff continuing education and providing clinical trainings
Clinical Coordination	● Coordinate with outside providers and care transitions ● Carry clinical history among disparate systems ● Leverage the role and network to help participants access appropriate care
Medical and Psychiatric Co-Integration	● Function as medical care coordinators and general practitioners (GP) for participants who cannot or are unwilling to access traditional medical care ● Triage various medical concerns, administer medical treatments, provide first aid and wound care, and bridge prescriptions for medical medications

### Case Example:

A woman in her 40’s was originally referred to IMT in the context of ongoing homelessness, unclear diagnosis, and years of transferring between homeless shelters due to violent incidents. The participant was generally calm and polite, but at times was noted to be either elevated and outgoing or angry and hostile. In many instances, she also presented as confused or paranoid, and weeks later would appear to have no recollection of interactions during that time. There were some reports of possible seizures, but the participant’s insurance would change mysteriously, slowing down the process of obtaining an evaluation. The team was able to facilitate placement of this participant into a shelter with robust on-site support, which helped them gather information about her situation. Among the many odd occurrences, shelter staff were able to provide video footage of the participant on the roof at night, of which the participant had no recollection. This led the team and the participant to understand that she was experiencing dissociative identity disorder (DID). The team contacted a DID specialist, who helped build a better understanding of the diagnosis and therapeutic approaches to treatment. Over time, the team also worked with the participant to engage in medical care, which involved communicating with the participant’s outside medical providers to provide context for her incomplete and contradictory medical history. This ultimately made it possible for the participant to complete a full neurological evaluation, confirming a diagnosis of temporal lobe epilepsy, and the participant was placed on appropriate anti-seizure medication. Since that time, the participant’s amnestic episodes have decreased significantly, and she has made progress in integrating the different aspects of her identity. She is now living stably in supportive housing, manages her own medications, and has not been involved in any significant altercations in several years. As this case study suggests, the IMT psychiatrist often plays a critical role in determining medical versus psychiatric symptoms which are often complex and overlapping and must act with tenacity to unify social service and medical providers in pursuing adequate medical and psychiatric care for participants who may present with atypical and often puzzling and long untreated symptoms, and under the IMT model, have the time and resources to do so.

### Principles and Core Components of Intensive Mobile Treatment

IMT uses a person-centered, recovery-oriented approach, focusing first on building a treatment relationship and addressing immediate survival needs, as well as on hope and growth in moving gradually towards recovery. Even in its fundamental language, IMT focuses on participants as the core of the team, choosing to use the word “participant” rather than “client” or “patient” to highlight our work together as a partnership. Psychiatric diagnosis and treatment are seen as one of many tools available to help individuals attain a better quality of life. In this section, we will seek to elucidate several core components of these treatment strategies (Table 4).

### Social Justice and Advocacy

Social justice and anti-racism form the basis of all IMT work. We recognize these as not only guiding principles, but also as an ongoing process of learning and growth as a program to provide the best services possible to our participants. As such, we seek to maintain a perspective of humility and to approach our work with curiosity, openness, and without judgment. Staff operate from an awareness of their own perspectives, biases, and experiences and apply this in both engaging with participants and recognizing possible barriers to engagement. These practices are also utilized in treatment planning, team meetings, and supervisory discussions.

Supervision provides a forum where staff can explore how race, sexual orientation, gender identity, and socioeconomic status–intersectionality–affect their relationships with participants and other team members. It is also a forum to explore how our own biases may impact our work. In both supervision and in team meetings, staff are also encouraged to discuss the impact of social factors on participant treatment plans and strategies for overcoming systemic barriers. This allows team members to both consider new strategies for approaching treatment and to employ more conscious ways of relating to others.

Because of the stigma associated with physical and mental illnesses, IMT participants experience significant and pervasive institutional inequality. Staff are encouraged to speak openly in team meetings about how we can address these issues and respond to participant needs most effectively. Staff are also encouraged to consider a participant’s race when engaging emergency services during crises, or how a staff member’s intersectionality may impact a participant’s willingness to engage with team members, as well as with systems.

The following case example illustrates an instance of a team changing its approach after considering a participant’s intersectionality.

The team had been taking a black male participant for haircuts in a Harlem barber shop. He expressed feeling infantilized by having a young white female case manager accompany him to a black-owned barber shop. The case manager listened to his concerns, brought them back to the team, and proposed that they give cash directly to the participant to pay for the haircut, provided that he returned a receipt to the team. The team was hesitant to approve this plan because it deviated from the agency’s general practices. The case manager was able to explain how her age, race, and control of the money was experienced as paternalistic and negatively impacted the participant. She advocated that the participant had been coming to the IMT office regularly, actively participating in his treatment, and developing meaningful relationships with the team. Because of this, she argued that he should be trusted with this opportunity. The team approved the plan, and this small change allowed the participant to feel more empowered and strengthened his relationship with the team. The team realized the need to be more aware of the cultural nuances and sensitivities of their participants and committed to taking steps to better understand and respect their experiences.

### Object relations and the psychodynamic frame of IMT

There is an indispensable, fundamental relationship that develops between participants and staff which comes to form a psychodynamic frame of the IMT program. Participants generally are isolated in the community with few social supports and have a history of difficult and unfulfilling personal and therapeutic relationships. In contrast, IMT starts from an object-relations approach to care, in that it seeks to create deep, long-term treatment relationships in which participants can practice reciprocity, build interpersonal skills, and ultimately experience corrective attachment experiences that ripple out into other areas of their lives. IMT builds a therapeutic alliance by consistently showing up in participants’ lives, regardless of the chaos of their circumstances and despite intrapersonal conflicts or experiences with rejection, even with the team itself. The team approaches participants with curiosity rather than judgment and attempts to understand each participant beyond their difficulties and deficits. The team prioritizes demonstrating an unconditional positive regard for participants, in contrast to the chronically poor regard with which they have been viewed by various systems and treatment providers in the past. As trust is built, IMT acts as a consistent and safe holding environment within which psychological phenomena can be experienced and resolved. In other words, the long-term work of IMT can be seen within a psychodynamic psychotherapy framework in which transferences with various members of the team are used as tools to process emotions, behaviors, events, and identity. Similarly, countertransference can be shared and processed among all of the members of the team, which helps to maintain positive regard for the participant and reduce provider burnout (Gabbard, 2005).

Over the course of treatment, it is expected that participants will test the limits of the relationship. When this occurs, staff provide feedback, which can be better received and integrated when there is an existing foundation of trust between the participant and staff. Staff intuition regarding a participant’s ego strength and ability to engage in self-examination are essential psychodynamic principles that must be employed. Sometimes there will be ruptures in the treatment alliance, for which the team must provide opportunities for repair. For IMT participants–whose relationships have often been characterized by inconsistency, rejection, and loss–learning to repair a relationship is a crucial corrective experience, as illustrated in this example:

An IMT participant with a documented history of aggressive behaviors, particularly towards treatment providers and Department of Homeless Services police while residing in the shelter system was referred to IMT. He experienced significant challenges with interpersonal relationships, responded disproportionately to rejection, and pursued legal action against multiple agencies, successfully winning several cases. During IMT's work with the participant, he constantly threatened legal action, demanded immediate responses from staff that were not feasible, and utilized language towards staff that was perceived as disrespectful and triggering.

The team's approach was to create a space where he could express his emotions and challenging behaviors to staff to a certain extent. Staff allowed him opportunities to vent and attempted to collaborate on solutions to his concerns. Meetings were sometimes terminated due to unacceptable behaviors such as using derogatory language or making direct threats towards a staff member. The team maintained positive regard for him while processing the challenging relationship during team meetings and supervisions.

The team collectively followed a plan to maintain boundaries, which specified when to end meetings, how to redirect conversations to concrete tasks, and how to best give him control of decisions regarding his treatment. Over time, he gradually noticed that staff maintained respectful communication and provided consistent treatment, even in the face of his threats and inappropriate language. This consistent response allowed staff to push back gently when demands or behaviors became intolerable. Over the course of several years, the participant’s email complaints to the DOHMH and CUCS Executive Director decreased in frequency and intensity and ultimately ceased. He is now able to manage conflict directly with the team and no longer feels the need to contact external sources to resolve issues. This enabled him to work with the team to secure permanent housing, and he is now working on other goals such as improving the quality of his interpersonal relationships.

The therapeutic approach at IMT is also informed by a person-centered, positive psychology framework. In general, IMT participants have experienced traditional mental health systems as institutions that label them based on perceived deficits or symptoms, which in turn has led to mistrust of providers and contributed to an impoverished sense of self. At IMT, we state clearly to each participant that we want to know them as individuals. We approach each person with curiosity, identify strengths and skills, and work to understand where the participant can derive purpose and meaning. Whether in the community or at the office, IMT attempts to emulate aspects of the clubhouse model to create an atmosphere of acceptance and belonging. Meetings in non-traditional settings (at appointments, in transit, at restaurants, at residences) create new opportunities to explore strengths and goals. The team also makes time for experiences of celebration and joy, such as birthday parties or “soberversary” celebrations at the office, haircuts, shaves, or manicures at salons or barber shops, or trips to the zoo, baseball games, or other recreational activities.

### Harm Reduction and Risk

Harm reduction and motivational interviewing approaches are important tools used in serving IMT participants. These originate in the arena of substance use treatment, based on the observation that a significant subset of people suffering from addiction do not respond to treatments with strict rules and requirements (Marlatt, 1996; Miller & Rollnick, 1991; Ritter & Cameron, 2006). Both approaches utilize empathetic listening and reflections and avoidance of direct confrontation in favor of working to develop intrinsic discrepancy and cognitive dissonance, as well as strength-based approaches to support self-efficacy and optimism (Miller & Rollnick, 1991). IMT employs a similar ethos with participants, both in addressing substance use and in addressing problematic behaviors overall. Individuals referred to IMT typically have not responded to or have experienced trauma within traditional systems, and rigid, restrictive approaches result in rejection of the team or continued treatment stagnation. Approaches that are flexible in real time are critical to building a foundation of trust and collaboration between IMT providers and participants.

Embedded in IMT’s model is a nuanced approach to risk assessment. For all IMT participants, the team must be able to tolerate a higher level of risk than traditional treatment teams. High-risk patients commonly use threats as a form of communicating intense and intolerable affect. What differentiates IMT participants is not only the intensity and frequency of these threats, but also their tendency to reject teams that respond to their risky or chaotic behaviors in typically reactive ways. For example, an IMT participant may sever communication with the team if 911 is called for expressing suicidal or homicidal ideation.

One IMT team worked with a participant who was at risk of engaging in violent behavior and regularly made threats of using a gun to harm others when he felt unheard. Initially, the team took the threats literally, but after meeting with him for several weeks it became clear that these statements were unsubstantiated and that he did not have the ability to obtain a gun. These statements served as a means to prevent anticipated future rejection and disappointment, and as a reflection of his core belief that the team would inevitably fail him, just as all other previous relationships had. The team engaged the participant in a conversation about the implications of his statements. They listened to his concerns, explored the underlying issues that were driving his behavior, and developed a plan to address his needs. By prioritizing what was important to him, such as helping him write letters to the President, the team was able to begin building trust. Through this process, the participant learned that making violent statements was not an effective way to get his needs met, and in turn he developed more productive ways to communicate his needs. The team's individualized care plan allowed them to provide tailored support that helped the participant manage his risk factors and achieve better outcomes.

The relationship is key; teams come to know participants well enough to determine when the level of risk can be tolerated and alleviated without involving emergency services. IMT also does not typically discontinue access to controlled substances or medication assisted treatment for positive drug screens, evidence of diversion, or other “infractions”; these are understood as part of recovery from addiction and tolerated in service of the relationship and in respect for the inherently seesaw-like process of recovery.

On the other end of the spectrum, another subset of IMT participants appear quite well by ordinary evaluation, but in reality, they are unsafe in the community due to violence, self-harm or functional impairment. Again, our relationship and understanding of the participant is critical in being able to inform ourselves and other providers that the risk is higher than it appears. In this case, IMT mobilizes a higher intensity of advocacy for escalating to a higher level of treatment and must communicate to other providers the subtle changes in presentation that indicate greater symptom severity. This level of nuanced evaluation is typically not able to be achieved in the in- and outpatient structures in our current mental health system, as evidenced in this example:

The treatment team discovered that a participant was under evaluation in the psychiatric emergency department of a local hospital. This participant was homeless and she frequently utilized emergency rooms for shelter. Typically, she would express suicidal ideation, often in the setting of substance use, only to later deny suicidality when she desired to leave the hospital. The hospital staff often diagnosed the participant with substance use and “malingering.” However, based on interactions with the participant in the community for over a year, the team observed that this participant was experiencing symptoms of schizophrenia, including internal preoccupation and thought disorganization, which contributed to her poor self-care and hindered her ability to manage basic necessities like food, shelter, and diabetes care. These symptoms were often overshadowed in the emergency department by the participant’s symptoms of intoxication or her demands for food and clothing. The team shared collateral information with the emergency department physician, who worked with them to create a plan for admitting the participant to an inpatient unit. From there, the team coordinated a transfer to a specialty care unit at another hospital.

While clinicians may balk at assuming liability for these high-needs participants, these are shared risks which do not fall solely on the psychiatrist. Team supervisors and program directors also assume risk for participants, with substantial institutional support to address difficult cases. With the backing of supervision and clinical support, the agency’s legal team, and monthly agency high-risk meetings, we are able to address these cases with confidence.

### Social Approaches and Health Advocacy

A critical mandate of IMT is that it must work nimbly within the existing systems and communicate with persistence and clarity with non-IMT service providers. IMT is able to advocate for our participants within systems that are traditionally overburdened, understaffed, and under-resourced, and help coordinate and optimize care for the already difficult-to-engage individual. Creating safety, contingency, and behavioral plans helps other treatment teams work with our participants, and collaboration between IMT and other city providers keeps participants in housing, out of hospitals, and free from involvement of law enforcement or emergency services.

For example, IMT in collaboration with a participant’s residence staff can foster a more nuanced understanding of their patterns of behavior so that 911 calls can be minimized. In one instance, an IMT participant living in a supportive housing residence frequently made alarming and provocative threats of violence towards housing staff when triggered by delusions that they were stealing his money. This behavior caused housing and security staff in the building to activate 911 frequently and request for the client to be assessed in the hospital. The IMT team was concerned that this pattern was re-traumatizing and increased his risk of exposure to police violence, especially due to his demographic profile as a tall black man. Further, this response was not yielding positive outcomes, as he would be quickly assessed and discharged from emergency rooms without any change in treatment. The IMT team was able to provide context around his behavioral patterns to the residential staff, noting that his threats were generally unsubstantiated. The team collaborated with his case manager to develop an individualized plan for assessing and intervening with the participant. The plan included specific instructions for building security, provided context as to how his behavior often unfolds, and gave further direction for what to do if the participant was unable to de-escalate. Additionally, it provided a script for housing staff to use when contacting NYPD or EMS to reduce the potential for negative or dangerous interactions.

If 911 must be called, IMT can be present to help intervene with police and emergency personnel using trauma-informed practices to minimize the risk of re-traumatization. If certain systems are not working for the participant, IMT scours the city for resources that may be more suitable or tolerable for their specific needs, and helps with the transition to the new system. Often extra assistance, accommodation, and accompaniment is needed for IMT participants to tolerate the stress of transitioning into new systems. IMT provides this level of support repeatedly until a goal is able to be reached. This can mean multiple housing transfers, many bouts of re-applying for benefits, obtaining and re-obtaining birth certificates, social security cards, and other documents, and working with legal systems to advocate in the cases of participants with legal involvement.

### Commitment to Fostering Effective Team Dynamics

IMT Teams are designed to have a low ratio of 1 staff to 3 participants. Team members share responsibility for all participants, with the intention that all play an active role in treatment decisions. This flattened hierarchy is a disruption of the top-down medical model and is a key component of IMT team dynamics and therapeutic decision making. Since traditional approaches have not worked for our participants, IMT is tasked with formulating alternative treatment strategies from various perspectives and areas of expertise. Since it is often difficult to predict what interventions will work for a given individual, each team member’s area of expertise and perspective is considered with equal weight and value. In contrast to the common ‘doctor-knows-best’ style of team dynamics, IMT psychiatrists collaborate as one part of a team, with openness to the fact that other team members may have equal or greater insights and strategies, even in domains that have been traditionally considered a doctor’s and a doctor’s alone, e.g., discussing medications. Flattening the team hierarchy empowers staff members to think creatively, beyond the bounds of their designated specialty. This distinctive collaboration within the team in turn creates a unique collaborative stance between the team and the participants. Only when everyone on the team shares a sense of empowerment and accountability for the treatment can we expect these dynamics to be reflected in the relationship with the participant.

**Table 4:** Core Components of IMT

Treatment is for anyone	Social Justice	The relationship is the core of the treatment	Accountability and longitudinal care
There is no diagnostic inclusion or exclusion Treatment is recovery oriented rather than illness based IMT makes every effort to eliminate traditional barriers	The team works towards awareness of their own biases and how it affects treatment Team members provide advocacy within traditionally racist and unjust systems	IMT maintains unconditional positive regard, in contrast to poor regard participants have experienced elsewhere There is a focus on the long-term work of building trust, learning to rupture and repair, rather than discontinue treatment when there is conflict	IMT stays involved even when people are incarcerated or in a long-term hospitalization IMT holds the participant’s history as the participant moves through other fragmented systems The team promotes secure attachments

Team Integration	The psychiatrist fills varied roles	Treatment is flexible, practical, person-centered and harm reducing	
There is a flattened hierarchy and shared decision making The team has a shared caseload and frequent team meetings Regular supervision allows space for reflection and processing The team can share countertransference and risk tolerance	Providers practice a flexible approach to prescribing The psychiatrist often integrates other medical treatments They provide psychotherapy and psychodynamic framing with the team The psychiatrist is involved in coordination and advocacy with outside providers and systems	The non-billing model allows for innovative approaches Service dollars allow the team to effectively meet immediate needs The team tolerates a higher level of risk, and practices flexible approaches to harm reduction There is no order in which problems must be addressed, and multiple goals can be addressed in tandem	

### Limitations, Challenges, and Avenues for Further Exploration

Up until this point, we have outlined the benefits of the flexible, creative approaches that are possible under the structure of the IMT model, which is, relatively speaking, well-funded in terms of human resources and service infrastructure. Here we explore challenges and areas requiring further optimization.

As a relatively new treatment model with fewer specific programmatic requirements than a model like ACT, there is a significant amount of heterogeneity among IMT teams. One area of heterogeneity is the staffing structure of teams. At our agency, there have already been several variations to our staffing structure which have resulted from accumulation of experience and rapid growth. Currently, most staff members work full time on one team of 27 participants, while the Program Directors, Assistant Programs Directors, and Peer Specialist Supervisors straddle two teams, or a total of 54 participants. This structure allows for more total staff on each team but does create several challenges for scheduling and time management. On the other hand, psychiatrists are funded at 2.5 days per week, which can be problematic as psychiatric issues can arise at any time.

Additionally, due to the high-risk nature of this work, IMT teams benefit from strong institutional support. The model we present above may be difficult to reproduce in resource-poor settings in which robust legal, fiscal, or administrative supports are not available.

Questions arising around the expandability of the IMT model involve the reliance on grants or other independent funding sources that do not require Medicaid billing. While this structure is possible in New York City, it is unclear whether it is transferable to places with differing priorities and resources. In New York City, IMT has expanded from one team in 2016 to 32 teams in 2022, and it is unclear whether this rate of growth is sustainable. The rapid addition of IMT teams has allowed for the treatment of greater numbers of New Yorkers with SMI, and has also resulted in staffing and space pressures, as well as concerns around dilution of team ethos and morale. Additionally, with the reduction in human services workers that occurred as a consequence of the coronavirus pandemic, the pre-existing shortage of mental health providers has been stretched further, resulting in significant hiring and retention challenges. Funding on the federal and state level intended to expand training and education of larger future generations of mental health practitioners and social service workers has not been realized and regardless will not address near-term staffing needs; thus, exploration of more sustainable solutions is greatly needed.

On a programmatic and team level, other issues will require further thinking in the future. Due to the complex scale of challenges participants often have faced, graduation or discharge from IMT teams is rare; however, as teams mature and participants engage with teams for longer periods of time, defining criteria for graduation and avenues for successful transition from IMT will need to be developed.

Finally, issues ubiquitous to the fields of mental health and social services also apply to IMT teams, including staff burnout/wellness, team building/morale, and staff development. How these issues will look in the specific environment of IMT will need further investigation.

## Conclusion

IMT is a novel treatment modality which builds on practices of other community treatment teams and is intended to address the needs of individuals who have fallen through the cracks of the mental health, housing, and criminal justice systems. This model exists only in New York City and is funded through contracts with NYC Department of Health. IMT has been successful at engaging people who have experienced significant trauma and systemic discrimination, and who have not been served by other existing treatment modalities. Our model prioritizes the formation of therapeutic relationships, and we use these long-term relationships to help participants change and grow over time. We are hopeful that efficacy and outcomes data demonstrating impact on hospitalizations, housing placements, episodes of incarceration, and other measures will be available in the near future.
